# Improved Canker Processing and Viability Droplet Digital PCR Allow Detection of *Erwinia amylovora* Viable Nonculturable Cells in Apple Bark

**DOI:** 10.3390/microorganisms12020376

**Published:** 2024-02-12

**Authors:** Bidhan Chandra Dhar, Ricardo Delgado Santander, Srđan G. Aćimović

**Affiliations:** 1 Alson H. Smith Jr. Agricultural Research and Extension Center, School of Plant and Environmental Sciences, Virginia Polytechnic Institute and State University, 595 Laurel Grove Rd, Winchester, VA 22602, USA; bidhandhar@vt.edu; 2 Irrigated Agriculture Research and Extension Center, College of Agricultural, Human and Natural Resource Sciences, Washington State University, Prosser, WA 99350, USA; r.delgadosantander@wsu.edu

**Keywords:** live cell quantification, DNA extraction, maceration, propidium monoazide, PMAxx, viability droplet digital PCR (v-ddPCR), VBNC

## Abstract

The bacterium *Erwinia amylovora* causes fire blight and continues to threaten global commercial apple and pear production. Conventional microbiology techniques cannot accurately determine the presence of live pathogen cells in fire blight cankers. Several factors may prevent *E. amylovora* from growing on solid culture media, including competing microbiota and the release of bacterial-growth-inhibitory compounds by plant material during sample processing. We previously developed a canker processing methodology and a chip-based viability digital PCR (v-dPCR) assay using propidium monoazide (PMA) to bypass these obstacles. However, sample analysis was still time-consuming and physically demanding. In this work, we improved the previous protocol using an automatic tissue homogenizer and transferred the chip-based v-dPCR to the BioRad QX200 droplet dPCR (ddPCR) platform. The improved sample processing method allowed the simultaneous, fast, and effortless processing of up to six samples. Moreover, the transferred v-ddPCR protocol was compatible with the same PMA treatment and showed a similar dynamic range, from 7.2 × 10^2^ to 7.6 × 10^7^ cells mL^−1^, as the previous v-dPCR. Finally, the improved protocol allowed, for the first time, the detection of *E. amylovora* viable but nonculturable (VBNC) cells in cankers and bark tissues surrounding cankers. Our v-ddPCR assay will enable new ways to evaluate resistant pome fruit tree germplasm, further dissect the *E. amylovora* life cycle, and elucidate *E. amylovora* physiology, epidemiology, and new options for canker management.

## 1. Introduction

The bacterium *Erwinia amylovora*, causing fire blight in rosaceous plants, continues to put at risk commercial apple and pear production in the United States and other countries worldwide [1,2]. A critical stage of fire blight disease is canker formation in perennial wood tissues, generally towards the end of the infection cycle. Under favorable conditions, fire blight cankers expand and girdle the organs where they form, blocking the flow of water and nutrients and killing the plant tissues above the canker. This leads to the death of branches or the entire tree when cankers form on the central leader, trunk, or rootstock [3,4]. Apart from the damage, *E. amylovora* overwinters in cankers and re-activates as the host renews growth in the next season. With conducive weather conditions, active cankers ooze out *E. amylovora* cells and act as primary inoculum sources for new outbreaks [1,3].

The biology of the pathogen in cankers, particularly conditions favoring canker formation and enabling *E. amylovora* survival in these structures, have been understudied, partly due to the lack of appropriate methodologies. The high content of polyphenols, tannins, and other PCR-inhibiting and bacterial-growth-inhibiting compounds, together with saprophytic microbiota, make it challenging to consistently isolate and quantify the pathogen from cankers using traditional methodologies. The toxic compounds and the natural microbiota present in cankers, released during plant material processing and co-cultured with *E. amylovora* on isolation media, may affect *E. amylovora* growth and make it difficult to extract and/or amplify DNA for molecular diagnostics. Environmental factors such as low or high temperatures, sample dehydration, and poor preservation conditions might also affect *E. amylovora* culturability during isolations. Most molecular detection and quantification methods cannot distinguish live from dead cells. Cankers also differ in size, thickness, hardness, etc. These factors make canker sample processing and analysis difficult, often providing inconsistent results.

In a previous work, Santander et al. [5] optimized a canker sample processing method through hammering in the presence of diluted antioxidant maceration buffer and developed a viability digital PCR (v-dPCR) assay using the viability dye propidium monoazide (PMA) and the chip-based QuantStudio 3D digital PCR (QS3D dPCR) to detect and quantify live *E. amylovora* cells in pear and apple cankers [5,6]. PMA, which is a photoreactive dye used to detect viable microorganisms via PCR, covalently binds with DNA in the presence of visible light, making it unsuitable for PCR amplification. PMA cannot pass through intact membranes of live cells, so it mainly attaches to dead cells’ DNA and the PCR analysis of samples treated with the dye selectively detects live cells [7,8]. The key advantages of the developed methodology were the culture-independent selective detection and absolute quantification of live *E. amylovora* cells in cankers without the need for standard curves. Moreover, dPCR is less impacted by inhibitors in comparison to other PCRs. The developed protocol was used successfully to analyze *E. amylovora* population dynamics over time in apple, pear, and Asian pear cankers and assess potential links between the host resistance, weather conditions, and pathogen survival in cankers [9]. However, some drawbacks were associated with both the sample processing method and the chip-based v-dPCR. For instance, processing samples inside plastic bags via hammering is time-consuming, noisy, and labor-intensive. Moreover, the QS3D dPCR platform only accepts 24 samples per run; the chip loading is cumbersome and slow and requires meticulous training to avoid issues with chip sealing or sample distribution within the chip.

In this study, we improved our previous protocol by automating sample processing using a SPEX Geno/Grinder 2010 homogenizer and transferring the previous v-dPCR to BioRad’s new, more user-friendly QX200 droplet dPCR (ddPCR) platform. Additionally, we used this new protocol to determine *E. amylovora* distribution within and around the canker tissues. Canker processing with the homogenizer allowed the fast, effortless, and simultaneous analysis of up to six samples per run and improved the reproducibility and DNA extraction yields. Moreover, the chip-based QS3D dPCR assay transfer to BioRad’s QX200 ddPCR platform provided similar dynamic ranges with both technologies and the capacity to selectively quantify live *E. amylovora* cells in PMA-treated samples. Finally, the optimized protocol combined with traditional plate counts allowed the quantification of viable *E. amylovora* cells in tissues around the cankers and revealed the existence of viable but nonculturable (VBNC) cells of the pathogen in and/or around canker tissues. 

Although the improved protocol was optimized with apple cankers, it may be adapted to other host species and cultivars [9] and help shed light on unknown aspects of the *E. amylovora* life cycle, elucidate the epidemiology of *E. amylovora*, and open new possibilities for canker management.

## 2. Materials and Methods

### 2.1. Bacterial Strain, Culture Media, and Inoculum Preparation

All studies were conducted using the American *E. amylovora* strain *Ea273*, also used in our previous study [5]. The bacterial cultures were cryopreserved at −80 °C in 20% glycerol (*v*/*v*). *E. amylovora* was grown at 28 °C in/on LB medium [10] and/or Sucrose Nutrient Agar (SNA) [11]. *E. amylovora* isolation and plate counts from natural samples were performed on SNA amended with 21.6 mg L^−1^ natamycin (SNAN) [5,12] and/or on Crystal violet-Cycloheximide-Thallium nitrate (CCT) medium [13]. 

Bacterial inocula were prepared with overnight cultures of *Ea273* in LB at 28 °C with shaking (180 rpm). Cultures were concentrated via centrifugation at 10,000 rpm for 2 min and the pelleted cells were rinsed thrice with sterile 10 mM phosphate buffered saline (PBS) pH 7.4, adjusted spectrophotometrically to 10^9^ CFU mL^−1^, and diluted as required for different assays.

### 2.2. Plant Material Collection and Cryopreservation

Natural fire blight canker samples were obtained from ‘Honeycrisp’ and ‘Evercrisp’ apple trees in orchards in Berryville and Winchester, VA (USA). Cankers were cut with sterile pruning shears, excising the portion of each branch containing a canker plus at least 2–3 cm above and 2–3 cm below the canker. 

*E. amylovora*-free plant material came from the Alson H. Smith Jr. Agricultural Research and Extension Center at Virginia Polytechnic Institute and State University in Winchester, VA (USA). The lack of *E. amylovora* cells in these samples was confirmed using microbiological and molecular methods, following the EPPO standard PM 7/20 (3) for the analysis of asymptomatic plant material [14]. These samples were used as negative controls and to prepare *E. amylovora*-free plant macerates for different assays. 

Both canker samples and *E. amylovora*-free plant material were stored at −80 °C after being flash-frozen in a high-density polyethylene plastic Dewar flask with liquid nitrogen.

### 2.3. Plant Material Processing Optimization

We compared hammering as a canker homogenization method, used previously [5,6,9], with an automatic homogenization using a SPEX SamplePrep Geno/Grinder 2010 (Thomas Scientific, Swedesboro, NJ, USA). Briefly, bark pieces from *E. amylovora*-free ‘Honeycrisp’ apple tree branches were weighed, and each gram of bark tissue was separated into two 0.5 g subsamples. One subsample was mixed in a ratio 1:50 (*w*/*v*) with 0.1× antioxidant maceration buffer (AMB) in a resealable plastic bag (i.e., 0.5 g bark and 25 mL 0.1 × AMB) inoculated with *E. amylovora* at concentrations in the range of 7.5 × 10^3^–7.5 × 10^6^ CFU mL^−1^ and processed via hammering on a flat surface, as described previously [5,6]. Antioxidant maceration buffer (1×) contained 20.0 g polyvinylpyrrolidone (PVP 10), 10.0 g mannitol, 1.76 g ascorbic acid, 3.0 g reduced glutathione, and 1 L of PBS 10 mM, pH 7.2 [14]. The other subsample was mixed with 25 mL 0.1 × AMB and the same *E. amylovora* concentrations in a 50 mL polycarbonate vial (Ops Diagnostics, Lebanon, NJ, USA) containing three 7/11-inch-diameter (1.62 cm diameter) steel beads (Ops Diagnostics, Lebanon, NJ, USA). The vials were then placed in a Cryo-Block (Ops Diagnostics, Lebanon, NJ, USA) and ground in the Geno/Grinder for 5 min at 1630 rpm. Afterwards, 0.4 mL aliquots of the inoculated plant macerates were used for DNA extraction and *E. amylovora* cell quantification via ddPCR analysis. Each of the assayed conditions was tested in triplicate.

### 2.4. PMAxx Dye Treatment and DNA Extraction

The viability staining was performed with an improved version of PMA, PMAxx (Biotium Inc., Fremont, CA, USA), according to Santander et al. [5]. Briefly, 0.4 mL aliquots of plant macerates were mixed with 0.1 mL of 5 × PMA Enhancer for Gram-negative bacteria (Biotium Inc., Fremont, CA, USA) and 2.5 μL of 20 mM PMAxx in 2 mL tubes. The tubes were then incubated for 5 min under dark conditions, at room temperature, in a shaker at 150 rpm to allow the dye to penetrate the dead cells. For PMAxx photo-activation, the tubes were placed horizontally and half-dipped on ice in a shallow tray located 20 cm distance under two halogen lamps (500 W each) for 10 min, with the researcher gently mixing the tubes’ content every 5 min. After treatment, samples were pelleted via centrifugation at 14,500× *g* for 10 min, the supernatants were discarded, and the pelleted materials were stored at −80 °C or directly used for DNA extraction, according to PMAxx manufacturer’s instructions.

Unless stated differently, the DNA extractions of pelleted PMAxx-treated and non-PMAxx-treated samples in this study were carried out with the Plant DNeasy Mini Kit (Qiagen, Frederick, MD, USA), following the manufacturer’s instructions.

### 2.5. Comparison of Different Commercial and Manual DNA Extraction Methods

The dynamic range of DNA-based methods for bacteria quantification can be affected by the procedures used for DNA extraction. To ensure the highest DNA extraction efficiencies from apple bark, the plant material used in this study, we tested the DNA extraction efficiencies of seven commercial kits and three manual extraction methods as follows. Bark samples from *E. amylovora*-free ‘Honeycrisp’ tree branches were mixed with 0.1 × AMB and homogenized with the Geno/Grinder as described above. Because column-based kits may show different DNA extraction efficiencies depending on the bacterial concentration in the samples, separate aliquots of the macerates were inoculated with *E. amylovora* at 10^3^, 10^5^, and 10^7^ CFU mL^−1^. Each method was tested with the three *E. amylovora* concentrations. We tested the column-based kits—ReliaPrep^TM^ kit (Promega Corporation, Madison, WI, USA), E.Z.N.A.^®^ SP Plant DNA Kit (Omega Bio-tek, Inc., Norcross, GA, USA), E.Z.N.A.^®^ bacterial DNA Kit (Omega Bio-tek, Inc., Norcross, GA, USA), E.Z.N.A.^®^ Plant DS Mini Kit (Omega Bio-tek, Inc., Norcross, GA, USA), DNeasy Plant Mini kit (Qiagen, Frederick, MD, USA), NucleoSpin^TM^ Plant II (Macherey-Nagel Inc., Allentown, PA, USA), and NucleoSpin^TM^ Soil (Macherey-Nagel Inc., Allentown, PA, USA)—and three different manual DNA extraction protocols [15,16,17]. The commercial kits were used following the manufacturer’s recommendations, with a final resuspension of DNA in 0.2 mL of elution buffer.

Uninoculated negative controls were included to prove the absence of *E. amylovora* in the analyzed *E. amylovora*-free plant material. To evaluate the DNA extraction efficiencies, we assumed that ddPCR is able to detect 100% of the target DNA copies present in the sample after DNA extraction. We calculated the efficiency of DNA extraction for each method, with each of the assayed bacterial concentrations, as the quantified copies of target DNA obtained via ddPCR with respect to the inoculated *E. amylovora* cell concentrations before the DNA extraction, multiplied by 100. Then, we defined the DNA extraction efficiency of each method as the average % DNA extraction efficiency obtained with samples inoculated with each assayed *Ea273* concentration. Accordingly, 100% efficiency corresponds to ddPCR values coinciding with the bacterial concentrations present in the samples before DNA extraction.

### 2.6. Transfer of a Chip-Based dCPR to a ddPCR Platform

The ddPCR experiments were performed using the QX200 ddPCR System (BioRad, Hercules, CA, USA) and the same primers and probes from our previous work with the chip-based QS3D dPCR system [5]. Briefly, we combined the reverse primer Ams189R (5′-GGG TAT TTG CGC TAA TTT TAT TCG-3′) and the probe Ams141T (5′-[FAM]—CCA GAA TCT GGC CCG CGT ATA CCG—[TAMRA]-3′), designed by Pirc et al. [18], with the forward primer Ams06Kb (5′-AAT TGG TTC CGC TAT AAC TTG CAG-3′) designed in a previous work [5] to avoid dead-cell signal amplification during v-dPCR by increasing the amplicon size (from 74 bp to 627 bp) [2]. Each ddPCR reaction was carried out in a volume of 20 μL containing 1 × ddPCR Supermix for probes (No dUTP) (BioRad, Hercules, CA, USA, cat. 1863024), 900 nM forward and reverse primers, 250 nM probe (final concentrations), and 6 μL DNA sample. Uniform oil droplets were generated in a QX200 droplet generator (BioRad, Hercules, CA, USA), and 40 μL of the mixture of sample plus oil was transferred to a 96-well PCR well plate and amplified in a C1000 Touch^TM^ Thermal Cycler (BioRad, Hercules, CA, USA, with a 96-well fast reaction module #1851196). 

For the thermal cycling conditions, we evaluated 2- and 3-step ddPCR cycles. Given the similar results and the speed of the 2-step thermal cycles, we chose this option for all the assays. Moreover, we assessed the optimal annealing temperature under the assayed conditions in a gradient ddPCR from 55 °C to 65 °C, resulting in a slightly higher temperature than in the previous work [5]. The optimized thermal cycling conditions were 94 °C for 10 min to activate the hot-start Taq polymerase, followed by 40 cycles of denaturation at 94 °C for 30 s (ramp rate of 2 °C/s) and annealing/extension at 61 °C for 2 min and a final signal stabilization cycle at 98 °C for 10 min. A hold step at 4 °C was also included to preserve samples until analysis. 

After amplification, fluorescence signals were read in the QX200 Droplet Reader (BioRad, Hercules, CA, USA) and analyzed with BioRad’s QX200 QuantaSoft Software Standard Edition (version 1.2). The threshold line differentiating positive from negative calls was determined automatically by the software and manually adjusted when necessary, using the signals in no-template controls as a guide. The software’s output data included the number of copies per μL, the number of droplets qualified for quantification, and the number of positive and negative calls for target DNA amplification, as well as other parameters linked to quantification and the quality assessment of the analysis. The minimum number of accepted droplets for quantification was 8000. Based on the analysis of *E. amylovora*-free plant material DNA, only reactions with positive call numbers above seven were considered positive for *E. amylovora* detection.

Quantitative ddPCR data were used to calculate *E. amylovora* cell concentrations in natural samples, taking into consideration dilution factors, DNA extraction efficiency, etc.

### 2.7. Correlation between ddPCR Data and Plate Counts 

The correlation between ddPCR data and bacterial concentrations in artificially inoculated samples with *E. amylovora* was determined via linear regression analysis using data from two independent assays conducted in triplicate. For each of the tested bacterial concentrations (from 10^2^ to 10^8^ CFU mL^−1^), the assay’s coefficient of variability (%CV) was computed by dividing the SD by the mean of the repetitions and multiplying by 100. Based on Pavšič et al. [19], the lower detection and quantification limits were defined as the lowest concentration at which all replicates were positive for detection and the lowest concentration exhibiting positive detection in all replicates with a %CV equal to or below 25%, respectively.

### 2.8. Selective Detection of Viable E. amylovora Cells Using PMAxx and the QX200 ddPCR System

The capacity of BioRad’s QX200 ddPCR platform to discriminate between live and dead *E. amylovora* cells after viability staining with PMAxx was tested in two different assays. First, to demonstrate the similar ability of v-ddPCR (treatment with PMAxx followed by ddPCR) and ddPCR to quantify suspensions composed of live cells, we inoculated *E. amylovora*-free ‘Honeycrisp’ bark macerates with increasing *E. amylovora* live cell concentrations from 7.4 × 10^3^ to 7.4 × 10^6^ CFU mL^−1^. Sub-aliquots of each bacterial concentration were either treated with PMAxx followed by DNA extraction or subjected to DNA extraction directly, and the quantification data, obtained via v-ddPCR and ddPCR, respectively, were compared using linear regression in an assay performed in triplicate.

In a second experiment, the capacity of v-ddPCR to discriminate live *E. amylovora* cells in the presence of dead cells was assayed as follows. Dead *E. amylovora* cell stocks were obtained through exposure to 85 °C for 30 min. The absence of live bacteria in the dead cell suspensions was checked via spread plate using LB agar. *E. amylovora*-free ‘Honeycrisp’ bark macerates containing 10^6^ dead *E. amylovora* cells mL^−1^ were inoculated with live *E. amylovora* concentrations ranging between 7.4 × 10^3^ and 7.4 × 10^6^ CFU mL^−1^. Sub-aliquots of each sample were either subjected to DNA extraction and total cell numbers quantified through ddPCR or stained with PMAxx before DNA extraction and live cells quantified via v-ddPCR. The correlation between v-ddPCR data and plate counts in the presence of dead cells was evaluated through linear regression in an assay performed in triplicate.

### 2.9. Analysis of E. amylovora Total, Live, and Culturable Cell Populations in Fire Blight Cankers and the Peripheric Symptomless Bark Areas

A total of 9 natural fire blight apple cankers (3 from ‘Honeycrisp’ and 6 from ‘Evercrisp’ trees) were collected in June 2022 and cryopreserved at −80 °C, as described above. Each canker in the sample had determinate margins. 

**Figure 1:**
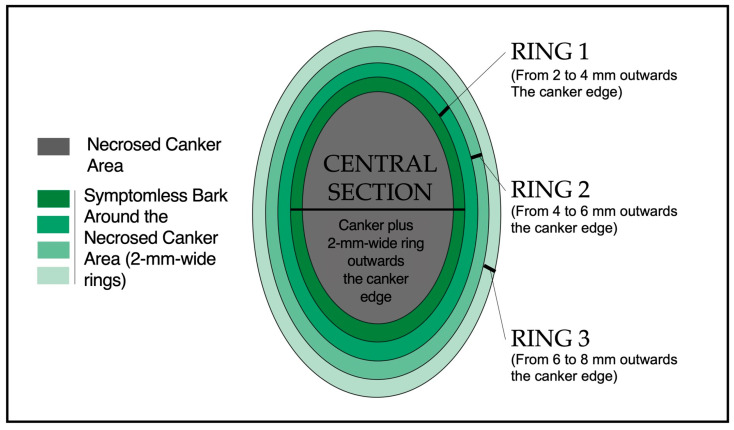
Canker sample sectioning. Each canker was sectioned in the central, necrosed canker area plus 2 mm around the canker and three concentric 2 mm wide rings around the central canker section (rings 1–3). Each section was processed individually for total, viable, and culturable cell counts.

For sample analysis, canker samples stored at −80 °C were allowed to defrost at room temperature for 10–15 min. To determine *E. amylovora* cell concentrations within the canker area, a central section delimited by the necrosed tissues plus 2 mm width symptomless bark area outwards the canker edge was excised aseptically and processed as described above (Figure 1). Additionally, we assessed *E. amylovora* concentrations in three 2 mm wide concentric ring areas surrounding the central section (Figure 1). Ring 1 comprised the bark area located 2 mm to 4 mm surrounding the central section. Ring 2 comprised the bark area located between 4 mm and 6 mm surrounding ring 1. Ring 3 comprised a third 2 mm wide ring-shaped section surrounding ring 2, i.e., 6 mm to 8 mm from the central section (Figure 1).

After the delimiting of the ring sections in each canker sample with a sterile scalpel, the bark and vascular cambium covering the sections were excised, weighed, and mixed with ice-cold 0.1 × AMB in a ratio of 1:50 (*w*/*v*). For the analysis of large cankers requiring 0.1 × AMB volumes exceeding the 50 mL polycarbonate grinding vial volume, 2–15 mL 0.1 × AMB buffer was added to the grinding vial for initial sample homogenization in the Geno/Grinder. The remaining volume of buffer (50 mL per gram of canker) was mixed with the homogenized sample afterwards. Sample macerates were kept on ice for no more than 30 min until use. 

For cell count analysis, sample macerates were mixed thoroughly and sub-aliquoted to perform (i) total cell counts via DNA extraction and ddPCR; (ii) viable cell counts via PMAxx viability staining, DNA extraction, and ddPCR (v-ddPCR) (see Section 2.4); and (iii) culturable cell counts via serial dilution and spread plate on SNAN and CCT media. When required, samples for ddPCR and v-ddPCR were diluted to reach target copy concentrations inside of the ddPCR quantification range.

### 2.10. Statistical Analysis

All the statistical analyses in this work were performed with GraphPad Prism 9 for MacOS (version 9.5.0). Differences between two groups or three or more groups of data following Gaussian distributions were assessed using Student’s *t* tests and ANOVA analysis, respectively. Bacterial population data were normalized via log transformation and analyzed using the parametric tests mentioned above. Datasets following non-normal distributions even after transformation through different methodologies were analyzed using Mann–Whitney U tests (comparisons of two datasets) or Kruskal–Wallis tests (comparisons of three or more datasets). The linear relationship between ddPCR and plate count data, or between ddPCR and v-ddPCR, was assessed using linear regression analysis. In all cases, the significance level considered for the analyses was set to 5%.

## 3. Results and Discussion

### 3.1. Automated Canker Homogenization Improves Recovery of E. amylovora from Cankers

**Figure 2:**
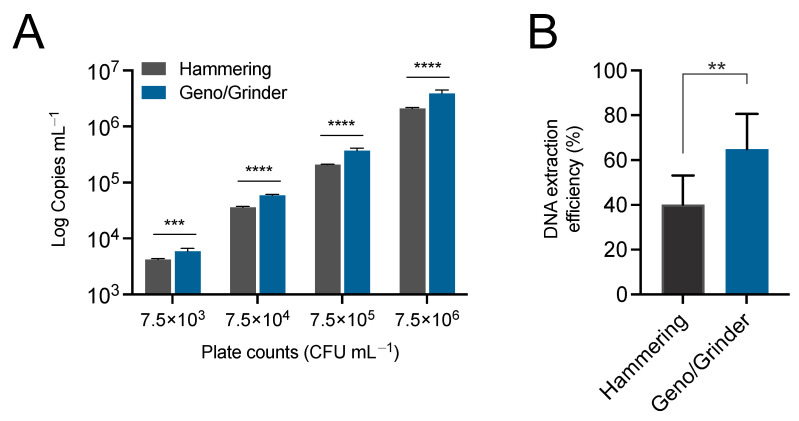
Effect of the plant material processing method on *Erwinia amylovora* ddPCR quantification and DNA extraction efficiency. Apple bark macerates obtained via hammering or using an automatized SPEX SamplePrep Geno/Grinder 2010 (in the charts, Geno/Grinder) were artificially inoculated with *E. amylovora* suspensions at different concentrations and analyzed through ddPCR. Charts show quantitative cell concentration data (**A**) and DNA extraction efficiencies (**B**) obtained after homogenization using the two methods. Error bars represent the standard deviation (SD). Asterisks denote significant differences based on post-hoc Šídák’s multiple comparison tests (**A**) or a Mann–Whitney U test (α = 0.05). **, *p* < 0.01; ***, *p* < 0.001; ****, *p* < 0.0001.

Sample homogenization methods impact subsequent analytical processes, from pathogen isolation to DNA yields [20,21]. Good tissue homogenization is necessary because pathogens do not usually distribute evenly in the sample. Ideally, a good sample processing method should allow the homogeneous release of the pathogen to the maceration buffer. This ensures proper detection through microbiological methods and enhances the capacity of DNA extraction reagents to reach all the pathogen cells equally. In our previous works, the processing of canker samples was carried out via hammering inside plastic bags [5,6]. Although reliable, this method is time-consuming, laborious, and noisy and requires physical strength, especially when analyzing large batches of samples. In such circumstances, the exhaustion of the person carrying out the task might contribute to incomplete or inefficient sample processing. In this study, we compared canker tissue homogenization via hammering and using an automatic homogenizer (Figure 2).

Although both methods showed high reproducibility of results (low variation between replicates), sample processing using the Geno/Grinder allowed better detection of *E. amylovora* cells using ddPCR (*p* ≤ 0.0009), on average 0.22 log-units higher. This corresponds to about a 66.4% increase in the detected pathogen cell numbers in samples homogenized automatically compared to hammered samples (Figure 2A). This improvement was especially higher (up to an 85.3% increase) when analyzing bark macerates containing the highest *E. amylovora* concentration (7.5 × 10^6^ CFU mL^−1^) (*p* < 0.0001).

The overall DNA extraction efficiency in samples processed with the Geno/Grinder (64.9%) also was significantly higher (*p* = 0.0023) than in hammered samples (40.2%) (Figure 2B). The highest DNA extraction efficiencies were obtained when analyzing bark samples containing low or moderately low *E. amylovora* concentrations (e.g., 10^3^–10^4^ CFU mL^−1^), regardless of the assayed sample processing method. Processing the same types of samples with the Geno/Grinder enabled DNA extraction efficiencies up to 30.4% higher than via hammering (Figure 2B).

The better detection and DNA extraction efficiency values in samples homogenized automatically were probably linked to the overall lower particle sizes obtained with the Geno/Grinder. Different authors [22,23] have reported a strong correlation between particle size and the DNA yields in plant samples. In their case, the higher DNA yields in small tissue particles were linked to the higher surface area exposed to the DNA extraction reagents. In our case, the obtained results could be explained by both a more efficient release of *E. amylovora* cells to the maceration buffer and the higher capacity of the DNA extraction reagents to act on *E. amylovora* cells trapped within smaller rather than larger particles of plant material when processed with the Geno/Grinder.

### 3.2. Column-Based Commercial Kits Provide Higher DNA Extraction Efficiencies than Manual Protocols 

**Figure 3:**
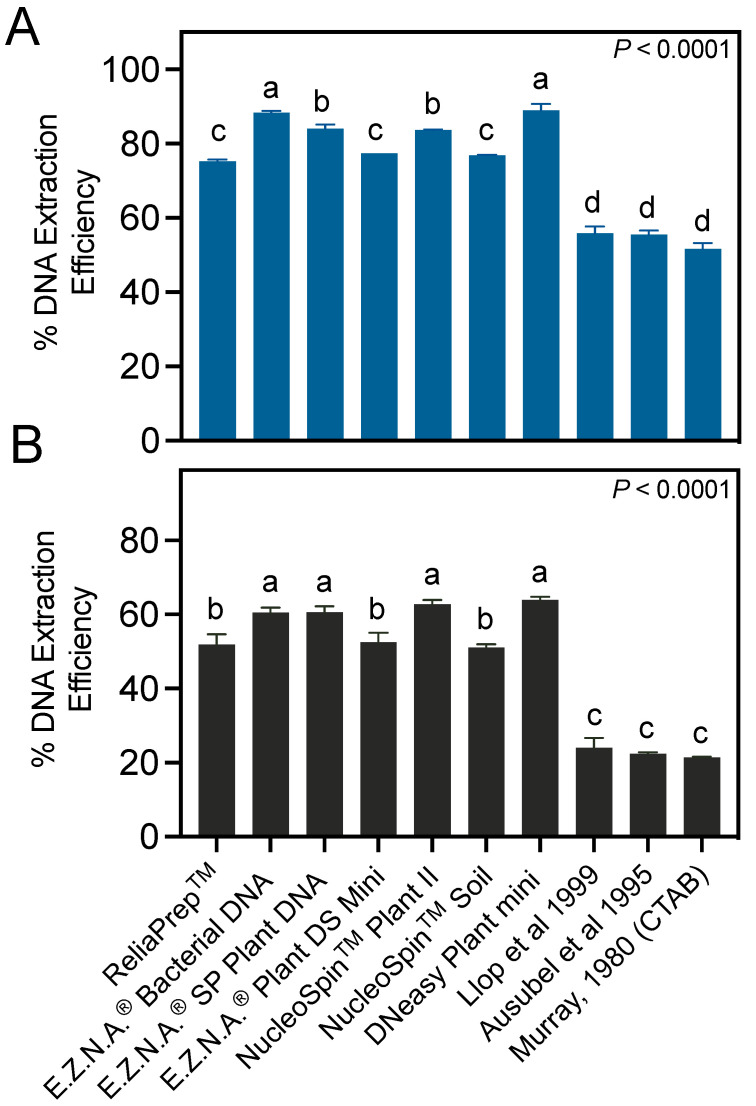
Efficiencies of different commercial kits and manual methods in DNA extractions of pure bacterial cultures (**A**) and artificially inoculated ‘Honeycrisp’ apple bark macerates (**B**). Columns indicate the average DNA extraction efficiencies (%) from ≥2 independent experiments and the error bars show the SD. The *p*-values on the upper right corner of each graph summarize the outcome of a one-way ANOVA analysis. Different letters indicate statistically significant differences between pairs of DNA extraction methods, assessed using Tukey’s post-hoc tests. The methods by Llop et al. 1999, Ausubel et al. 1995 and Murray 1980 correspond to references [15,16,17].

When DNA is used as an indirect method to quantify pathogen cells in samples, an important element conditioning the pathogen detection limit is the efficiency of DNA extraction. Too-low DNA extraction efficiencies may lead to underestimating the target DNA copies in the sample, favoring false negative detections. In this study, we compared the DNA extraction efficiencies of seven different column-based commercial kits and three manual extraction methodologies used in other works (Figure 3). 

One-way ANOVA analyses revealed significant differences between the assayed DNA extraction protocols, regardless of the type of sample analyzed (*p* < 0.0001) (Figure 2). In pure bacterial culture samples (Figure 3A), the highest DNA extraction efficiencies were obtained with the E.Z.N.A Bacterial DNA and DNeasy Plant Mini Kits, which provided similar efficiencies (*p* = 0.9989), around 88%. These values were significantly higher than those obtained with the other methods (*p* ≤ 0.0142). The DNA extraction efficiencies of the commercial kits were 20.8–34.5% higher than of those obtained through manual procedures (*p* < 0.0001), which were around 54.3% regardless of the manual protocol utilized (*p* ≥ 0.2646) (Figure 3A). 

All the DNA extraction methods performed up to 37.8% better with pure bacterial suspensions (Figure 3A) than with plant macerates artificially inoculated with *E. amylovora* (Figure 3B). Among the kits, the E.Z.N.A Bacterial DNA, E.Z.N.A SP Plant DNA, NucleoSpin Plant II, and DNeasy Plant Mini Kits provided the highest extraction efficiencies, of around 62% (*p* ≤ 0.0004) (Figure 3B). The DNA extraction efficiencies obtained with the column-based kits were 27.0–42.6% higher than those obtained through manual procedures (Figure 3B) (*p* < 0.0001). The manual method’s DNA extraction efficiencies were similar regardless of the assayed protocol (*p* ≥ 0.6739), around 22.6%. 

The success of pathogen DNA extraction from plant samples depends on different variables. Some plant species, cultivars, or tissues accumulate more polysaccharides, polyphenols, and secondary metabolites than others. These molecules interact and combine with nucleic acids and interfere with DNA extraction, reducing both the DNA yields and quality [24,25,26]. Kits and manual protocols allowing efficient DNA extractions with one type of plant material might be unsuitable for analyzing other sample types [20,22]. For example, in our experience, methodologies allowing efficient *E. amylovora* DNA extractions from apple bark provide much lower DNA extraction efficiencies with other host species and even cultivar [5,6,9]. Leaf DNA extractions usually provide better DNA yields than when attempting isolations from more lignified tissues like the bark of old branches or roots [27]. Even the age of the examined tissues may affect the quality of DNA extractions [28]. Other variables that we did not explore but that probably also impacted our DNA extraction efficiency values are the DNA integrity and purity obtained with each method. DNA degradation during sample processing and the co-isolation of sample components with the DNA have been shown to reduce the DNA extraction efficiency in studies with different types of samples [20,29,30]. Finally, bacterial concentrations in the sample also impact the DNA extraction efficiencies. In our experience, bacterial concentrations around 10^8^ CFU mL^−1^ or higher in macerates usually lead to very low DNA extraction efficiencies. For this reason, sample dilution is recommended when high bacterial concentrations are expected (large canker sizes i.e., weights). 

### 3.3. QX200 Droplet dPCR Provides Similar Quantification Dynamic Range Compared to the Chip-Based QS3D dPCR

**Figure 4:**
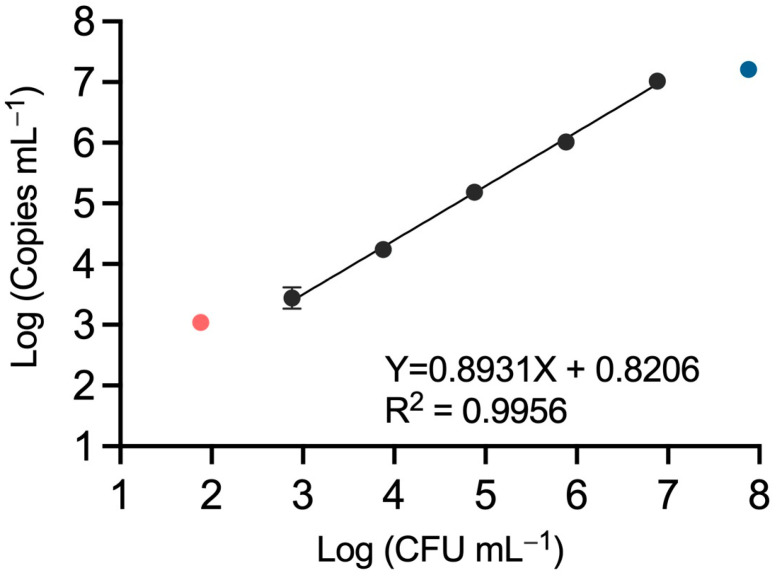
Linear regression analysis of *E. amylovora* culturable cell counts and QX200 ddPCR quantification data. Each dot corresponds to mean values of an experiment performed in triplicate. Error bars show the SD. Absent error bars indicate SD values smaller than the represented symbols. The solid line indicates the linear regression best fit through the dPCR dataset points. Black dots indicate *E. amylovora* concentrations within dynamic range. The blue dot indicates a cell concentration out of the dynamic range but reliably detected via ddPCR. The red dot shows a cell concentration out of the dynamic range, for which only some of the replicates were positive for *E. amylovora* detection.

In our study, we transferred a protocol to the QX200 ddPCR system that was designed for *E. amylovora* quantification using the chip-based QS3D dPCR platform [5]. A linear regression analysis of the relationship between plate counts and the copies mL^−1^ obtained using ddPCR showed a strong correlation (R^2^ = 0.9956) between BioRad’s QX200 ddPCR data and *E. amylovora* plate counts when analyzing bark macerate samples containing between around 10^3^ and 10^7^ CFU mL^−1^. Higher and lower *E. amylovora* concentrations led to the underestimation and overestimation of the pathogen populations, respectively (Figure 4).

The observed dynamic range of quantification, from around 10^3^ to 10^7^ copies mL^−1^, was equivalent to the one obtained with the chip-based QS3D dPCR technology [5]. Some of the sample replicates containing less than 10^3^ CFU mL^−1^ were negative for *E. amylovora* detection, i.e., the number of positive calls in some of the samples containing 10^2^ or less CFU mL^−1^ was equal to or lower than those observed in *E. amylovora*-free plant material (Figure 4, Table 1). Finally, although all the samples containing around 10^8^ CFUs mL^−1^ were positive for *E. amylovora* detection (Table 1), these were out of the dynamic range of quantification (Figure 4). This means dilutions are necessary to quantify samples containing more than 10^7^ target copies mL^−1^ accurately. These results indicate that the transferred protocol from the QS3D chip-based dPCR to the droplet-based QX200 ddPCR did not change the dPCR performance [5].

Although narrow, the obtained dynamic range with the QX200 ddPCR fits the results reported using other dPCR platforms [32,33,34,35,36]. The dynamic range of most commercially available dPCRs is of four orders of magnitude, mainly because of the limited sample partitioning capacity of the current dPCR platforms. However, the high precision and sensitivity of dPCR, together with the lowest inhibition by sample components or the absolute quantitation without the need for calibration curves compared to qPCR, still make dPCR a convenient choice when available [34]. Progress in dPCR technology and sample partitioning is being made, and the release of new platforms allowing wider dynamic ranges comparable to those of qPCR will probably end up replacing qPCR in many applications [34,37,38].

### 3.4. The Transferred v-ddPCR Protocol Efficiently Discriminates between Live and Dead E. amylovora Cells 

**Figure 5:**
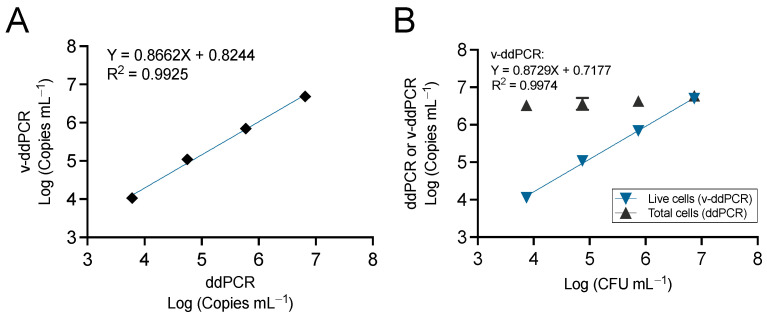
Performance of v-ddPCR, discriminating between live and dead *E. amylovora* cells in ‘Honeycrisp’ bark macerates. Correlation between ddPCR and v-ddPCR data in suspensions of live *E. amylovora* cells (**A**). Correlation between plate counts and v-ddPCR data in bark macerates containing mixtures of live cells at different concentrations plus 10^6^ dead cells mL^−1^ (**B**). Each dot corresponds to mean values of two independent experiments performed in triplicate. Absent error bars indicate SD values smaller than the represented symbols. Solid lines represent the linear regression best fit through all the ddPCR and/or v-ddPCR dataset points. The corresponding linear regression equations and coefficients of determination (R^2^) are also indicated.

Viability PCRs based on PMA allow the selective detection and quantification of live cells. However, different factors associated with the staining protocol, the PCR type, the primer design, the nature of samples, the pathogen species, etc. can lead to suboptimal discrimination between live and dead cells [39,40,41,42]. In a previous work, we optimized the viability staining protocol and the PCR amplicon size to analyze *E. amylovora* populations in canker samples using the chip-based QS3D dPCR [5]. To assess the efficacy of the same protocol with the QX200 ddPCR platform, we determined ddPCR total and v-ddPCR viable *E. amylovora* cell counts in bark macerates inoculated with live cells or mixtures of live and dead *E. amylovora* cells, using the protocols (Figure 5).

Both total cell quantification data from ddPCR and viable cell quantification data from ddPCR after PMA treatment (v-ddPCR) correlated well (R^2^ = 0.9925) when analyzing bark macerates inoculated with live *E. amylovora* cells (Figure 5A). In mixtures of live and dead *E. amylovora* cells, while v-ddPCR data showed a good correlation (R^2^ = 0.9974) with the inoculated *E. amylovora* CFUs mL^−1^ in the samples, total cell counts as per ddPCR showed stable numbers at around 10^6^ total cells mL^−1^ due to the 10^6^ dead cells mL^−1^ co-inoculated with live *E. amylovora* cells in each sample (Figure 5B). These results indicate that our previous protocol, optimized for the QS3D dPCR, is equally effective in selectively differentiating live from dead *E. amylovora* cells with the QX200 ddPCR platform.

### 3.5. Viable E. amylovora Populations in Tissues within and around Cankers Are Mostly Composed of VBNC Cells 

**Figure 6:**
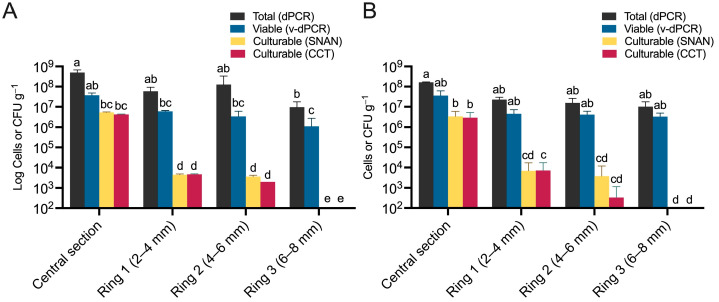
*E. amylovora* total, live, and culturable cell populations in natural fire blight cankers and tissues around cankers. The analyzed cankers were harvested from ‘Honeycrisp’ (**A**) and ‘Evercrisp’ (**B**) apple trees in June 2022. The charts show total (ddPCR), viable (v-ddPCR), and culturable cell counts (on SNAN and CCT media). Columns represent mean values of three and six different ‘Honeycrisp’ (**A**) and ‘Evercrisp’ (**B**) cankers, respectively, with error bars indicating the standard deviation (SD). Different letters denote statistically significant differences based on Tukey’s multiple comparison tests (α = 0.05).

Even though numerous studies have focused on characterizing cankers [43,44,45], accurate estimations of viable *E. amylovora* populations in these structures are scarce and mainly rely on culture-dependent approaches. In previous works, we successfully developed and applied a method for the molecular quantification of total and viable *E. amylovora* cells in cankers using dPCR [5,6,9]. In this work, we improved the canker sample processing method and tested the same viability-staining protocol using a different dPCR platform, the QX200 ddPCR. To assess the capacity of the improved protocol to analyze *E. amylovora* populations in cankers, we processed natural cankers from ‘Honeycrisp’ and ‘Ever Crisp’ apple trees and assessed potential differences between *E. amylovora* populations within the canker necrosed area and symptomless bark tissues surrounding the canker (Figure 6). 

Total and viable *E. amylovora* cell populations in all the analyzed cankers were slightly higher in the central necrosed area, around 10^8^ cells g^−1^ and 10^7^ cells g^−1^, respectively, than in the bark tissues around the cankers. In most cases, these differences were statistically non-significant (*p* ≥ 0.0629) except for ‘Honeycrisp’ cankers, which showed significantly lower total (*p* = 0.0010) and viable (*p* < 0.0001) *E. amylovora* cell concentrations in Ring 3, located furthest from the central canker area (Figure 6A). Total and viable *E. amylovora* cell numbers in Ring 3 averaged 9.7 × 10^6^ cells g^−1^ and 1.1 × 10^6^ cells g^−1^, respectively. 

Plate count data revealed similar culturable and viable cell concentrations in the central canker section (*p* ≥ 0.4358) (Figure 6A,B). However, we observed a sharp, progressive decrease in *E. amylovora* culturable cell populations correlating with the distance between the central section and the analyzed bark tissues around the canker (Figure 6). In both ‘Honeycrisp’ (Figure 6A) and ‘Evercrisp’ cankers (Figure 6B), *E. amylovora* culturable cell populations were around 3–5 × 10^6^ CFU g^−1^ in the central section but undetectable in the bark ring section farthest from the canker area (Ring 3) (*p* < 0.0001). These results contrasted with the viable cell population numbers assessed via v-ddPCR, which remained from 0.8 to 6.5 log-units higher than culturable cell counts assessed on culture media. Differences between viable and culturable populations reached the highest values in the tissues located in the bark of Ring 3, from 6 to 8 mm away from the canker edge (*p* < 0.0001) (Figure 6A,B). 

Differences between culturable and viable *E. amylovora* populations have been related to the entry of the pathogen into the viable but nonculturable (VBNS) state. Several stresses have been reported to induce this physiological response, including exposure to sublethal concentrations of copper, hydrogen peroxide, acetic acid, and chlorine and also starvation conditions in water or while surviving in/on insects [46,47,48,49,50,51,52,53]. Bacterial cells in this physiological state are alive and conserve certain metabolic functions but are unable to form colonies on solid microbiological media [54,55,56]. The presence of VBNC *E. amylovora* cells in infected bark tissues is also a plausible explanation for the inconsistent results between culture-dependent and molecular pathogen detection methods observed in different studies [45,57,58,59,60]. However, to our knowledge, we demonstrated, for the first time, the existence of viable but nonculturable, i.e., VBNC, *E. amylovora* cells in plant tissues of fire blight cankers.

Our study proved that v-ddPCR could discriminate between live and dead *E. amylovora* cells. Hence, our data from natural canker analysis indicate that at least part of the *E. amylovora* population enters the VBNC state during host tissue infection, especially in perimeter areas farther away from the canker edge, probably coinciding with the front line of infection. The induction of the VBNC state during host plant infections has also been described in *Ralstonia solanacearum* [61]. This response is probably linked to exposure to cytotoxic compounds released during host–pathogen interactions. In this regard, Mock et al. demonstrated that the oxidation of acetosyringone in the presence of hydrogen peroxide and peroxidase induces the VBNC state in *Pseudomonas syringae* pathovars [62]. Acetosyringone is a pattern-triggered immunity-related phenolic compound present in the apoplast of tobacco and other plant species [63], the oxidation of which also leads to growth inhibition in *Agrobacterium tumefaciens*, *Clavibacter michiganensis* subsp. *Michiganensis, Pectobacterium carotovorum* subsp. *Carotovorum,* and *Xanthomonas campestris* pv. *campestris* [63]. *E. amylovora* triggers an oxidative burst during compatible interactions [64,65], so exposure to oxidized acetosyringone or other phenolic compounds in the bark might explain the results we observed in this work. 

In addition to phenolic compounds, hydrogen peroxide and other cytotoxic metabolites released during *E. amylovora*–host interactions [64,65] might also trigger the VBNC response in this pathogen during infections. For instance, in a previous work, we demonstrated that hydrogen peroxide induced the VBNC state in the pathogen in vitro [49] and that mechanisms protecting cells against oxidative stress delay the entry of the fire blight pathogen into the VBNC state [66,67]. 

Finally, our results show that VBNC populations within and around cankers represent the predominant form of viable cells. For example, in the central section of the canker, culturable *E. amylovora* cells represent around 8.7–12.7% of the total viable cell population, 0.07–0.15% in rings 1 and 2, and below 0.03–0.09% in ring 3. In other words, the VBNC populations in the analyzed canker samples ranged from 87.3 to 91.3% of the total viable cell population in the central section and from at least 99.91 to 99.97% of the total viable cell population in Ring 3 (Figure 6).

## 4. Conclusions

Results in this study highlight the importance of optimizing DNA extraction procedures for the type of plant samples analyzed. The canker processing protocol and the transferred BioRad QX200 v-ddPCR assay optimized in this work allowed both an effortless and faster sample processing compared to the protocol developed previously [5,6] as well as the selective detection of live *E. amylovora* cells in vitro, with a dynamic range like that obtained with the chip-based QS3D dPCR. We demonstrated the technique’s usefulness, combined with plate counts, to explore the total, viable, culturable, and VBNC *E. amylovora* cell populations in cankers and the surrounding bark tissues. 

Some of the most important applications of dPCR in the plant science sector are in pathogen diagnostics, the detection of genetically modified plants, the characterization of transgenic lines, gene expression analysis, plant species detection, etc. [68]. The methodology for fire blight canker sample processing and ddPCR analysis optimized in this work has a significant potential for testing the effectiveness of bactericidal compounds in plants and conducting research on diverse aspects of the *E. amylovora* epidemiology, such as population dynamics preceding and during infection and the analysis of viable, culturable, and VBNC populations in insect vectors, reservoirs of the pathogen, etc. 

## Figures and Tables

**Table 1:** QX200 ddPCR analysis of serial tenfold dilutions of *E. amylovora* suspensions in ‘Honeycrisp’ bark macerates.

CFU mL^−1^	Replicate	Copies L^−1^	CV% ^a^	Copies mL^−1 b^	Positive Calls	Accepted Calls
7.60 × 10^7^	1	9992.19	13.10	1.67 × 10^7^	13,822	13,825
	2	10,983.49		1.83 × 10^7^	16,408	16,410
	3	8435.36		1.41 × 10^7^	12,502	12,505
7.60 × 10^6^	1	5996.34	4.02	9.99 × 10^5^	16,835	16,897
	2	6485.90		1.08 × 10^6^	16,789	16,857
	3	6345.67		1.06 × 10^6^	16,342	16,645
6.60 × 10^5^	1	546.99	10.83	9.12 × 10^5^	5744	11,596
	2	679.84		1.13 × 10^6^	5352	12,194
	3	627.34		1.05 × 10^6^	5281	11,093
7.60 ×10^4^	1	87.05	5.55	1.45 × 10^5^	725	9245
	2	97.14		1.62 × 10^5^	736	9287
	3	93.78		1.56 × 10^5^	729	9164
7.60 × 10^3^	1	10.12	9.61	1.69 × 10^4^	84	10,039
	2	11.60		1.93 × 10^4^	100	10,195
	3	9.68		1.61 × 10^4^	76	10,119
7.60 × 10^2^	1	2.06	20.44	3.43 × 10^3^	12	13,666
	2	2.12		3.53 × 10^3^	13	13,678
	3	1.43		2.23 × 10^3^	11	13,665
7.60 × 10^1^	1	0.72	13.21	1.20 × 10^3^	4	16,375
	2	0.56		9.33 × 10^2^	7	10,257
	3	0.7		1.17 × 10^3^	5	13,065
NTC ^c,d^	1	1.59	108.26	2.17 × 10^2^	7	14,902
	2	0.26		1.67 × 10^2^	3	14,484
	3	0.27		1.83 × 10^2^	4	14,327
NTC ^c^	1	0	173.21	0.00	0	13,127
	2	0		0.00	0	9385
	3	0.21		2.00	2	11,291
NTC ^c^	1	0	87.05	0.00	0	14,902
	2	0.08		1.00	1	14,484
	3	0.09		1.00	1	14,327

^a^ CV%, coefficient of variation. ^b^ *E. amylovora* concentration in the analyzed macerates, calculated using ddPCR data. ^c^ NTC, non-template control consisting of DNA extracted from *E. amylovora*-free ‘Honeycrisp’ bark macerates. ^d^ Some NTCs showed up to seven positive calls when analyzed through ddPCR. Because these samples were negative for *E. amylovora* detection based on culture-dependent methods and species-specific PCR [31], we established a minimum of eight positive calls necessary to consider a sample positive for *E. amylovora* detection.

## Data Availability

The data presented in this study are available on request from the corresponding authors.

## References

[1] van der Zwet T., Orolaza-Halbrendt N., Zeller W. (2016). Fire Blight History, Biology, and Management.

[2] Vanneste J. (2023). *Erwinia amylovora* (fireblight). CABI Compendium..

[3] Thomson S.V., Vanneste J.L. (2000). Epidemiology of fire blight. Fire Blight: The Disease and Its Causative Agent, Erwinia amylovora.

[4] Aćimović S.G., Santander R.D., Meredith C.L., Pavlović Ž.M. (2023). Fire blight rootstock infections causing apple tree death: A case study in high-density apple orchards with *Erwinia amylovora* strain characterization. Front. Hortic..

[5] Santander R.D., Meredith C.L., Aćimović S.G. (2019). Development of a viability digital PCR protocol for the selective detection and quantification of live *Erwinia amylovora* cells in cankers. Sci. Rep..

[6] Santander R.D., Gašić K., Aćimović S.G. (2022). Selective quantification of *Erwinia amylovora* live cells in pome fruit tree cankers by viability digital PCR. Methods Mol. Biol..

[7] Nocker A., Cheung C.-Y., Camper A.K. (2006). Comparison of propidium monoazide with ethidium monoazide for differentiation of live vs. dead bacteria by selective removal of DNA from dead cells. J. Microbiol. Methods.

[8] Nocker A., Sossa-Fernandez P., Burr M.D., Camper A.K. (2007). Use of propidium monoazide for live/dead distinction in microbial ecology. Appl. Environ. Microbiol..

[9] Santander R.D., Khodadadi F., Meredith C.L., Rađenović Ž., Clements J., Aćimović S.G. (2022). Fire blight resistance, irrigation and conducive wet weather improve *Erwinia amylovora* winter survival in cankers. Front. Microbiol..

[10] Bertani G. (1951). Studies on lysogenesis. I. The mode of phage liberation by lysogenic *Escherichia coli*. J. Bacteriol..

[11] Dye D.W. (1968). A taxonomic study of the genus *Erwinia*. I. The ‘amylocora’ group. N. Z. J. Sci..

[12] Pedersen J.C. (1992). Natamycin as a fungicide in agar media. Appl. Environ. Microbiol..

[13] Ishimaru C., Klos E.J. (1984). New medium for detecting *Erwinia amylovora* and its use in epidemiological studies. Phytopathology.

[14] (2022). EPPO standard on diagnostics: PM 7/20 (3) *Erwinia amylovora*. EPPO Bull..

[15] Llop P., Caruso P., Cubero J., Morente C., López M.M. (1999). A simple extraction procedure for efficient routine detection of pathogenic bacteria in plant material by polymerase chain reaction. J. Microbiol. Methods.

[16] Dobrowolski P., Ausubel F.M., Brent R., Kingston R.E., Moore D.D., Seidman J.G., Smith J.A., Struhl K. (1992). Short Protocols in Molecular Biology: A Compendium of Methods From “Current Protocols in Molecular Biology”.

[17] Murray M.G., Thompson W.F. (1980). Rapid isolation of high molecular weight plant DNA. Nucleic Acids Res..

[18] Pirc M., Ravnikar M., Tomlinson J., Dreo T. (2009). Improved fireblight diagnostics using quantitative real-time PCR detection of *Erwinia amylovora* chromosomal DNA. Plant Pathol..

[19] Pavšič J., Žel J., Milavec M. (2016). Assessment of the real-time PCR and different digital PCR platforms for DNA quantification. Anal. Bioanal. Chem..

[20] Demeke T., Jenkins G.R. (2010). Influence of DNA Extraction Methods, PCR Inhibitors and Quantification Methods on Real-Time PCR Assay of Biotechnology-Derived Traits. Anal. Bioanal. Chem..

[21] Rohde A., Hammerl J.A., Appel B., Dieckmann R., Al Dahouk S. (2015). Sampling and Homogenization Strategies Significantly Influence the Detection of Foodborne Pathogens in Meat. Biomed Res. Int..

[22] Holden M.J., Blasic J.R., Bussjaeger L., Kao C., Shokere L.A., Kendall D.C., Freese L., Jenkins G.R. (2003). Evaluation of extraction methodologies for corn kernel (*Zea mays*) DNA for detection of trace amounts of biotechnology-derived DNA. J. Agric. Food Chem..

[23] Moreano F., Busch U., Engel K.-H. (2005). Distortion of genetically modified organism quantification in processed foods: Influence of particle size compositions and heat-induced DNA degradation. J. Agric. Food Chem..

[24] Aboul-Maaty N.A.-F., Oraby H.A.-S. (2019). Extraction of high-quality genomic DNA from different plant orders applying a modified CTAB-based method. Bull. Natl. Res. Cent..

[25] Heikrujam J., Kishor R., Behari Mazumder P. (2020). The chemistry behind plant DNA isolation protocols. Biochemical Analysis Tools—Methods for Bio-Molecules Studies.

[26] Rucińska A., Olszak M., Świerszcz S., Nobis M., Zubek S., Kusza G., Boczkowska M., Nowak A. (2021). Looking for hidden enemies of metabarcoding: Species composition, habitat and management can strongly influence dna extraction while examining grassland communities. Biomolecules.

[27] Bahnweg G., Schulze S., Möller E.M., Rosenbrock H., Langebartels C., Sandermann H. (1998). DNA isolation from recalcitrant materials such as tree roots, bark, and forest soil for the detection of fungal pathogens by polymerase chain reaction. Anal. Biochem..

[28] Moreira P.A., Oliveira D.A. (2011). Leaf age affects the quality of DNA extracted from *Dimorphandra mollis* (Fabaceae), a tropical tree species from the cerrado region of brazil. Genet. Mol. Res..

[29] Claassen S., du Toit E., Kaba M., Moodley C., Zar H.J., Nicol M.P. (2013). A Comparison of the efficiency of five different commercial DNA extraction kits for extraction of DNA from faecal samples. J. Microbiol. Methods.

[30] Kemp B.M., Winters M., Monroe C., Barta J.L. (2014). How much DNA is lost? Measuring DNA loss of short-tandem-repeat length fragments targeted by the PowerPlex 16^®^ system using the Qiagen MinElute Purification Kit. Hum. Biol..

[31] Taylor R.K., Guilford P.J., Clark R.G., Hale C.N., Forster R.L.S. (2001). Detection of *Erwinia amylovora* in plant material using novel polymerase chain reaction (PCR) primers. N. Z. J. Crop Hortic. Sci..

[32] Dreo T., Pirc M., Ramšak Ž., Pavšič J., Milavec M., Žel J., Gruden K. (2014). Optimising droplet digital PCR analysis approaches for detection and quantification of bacteria: A case study of fire blight and potato brown rot. Anal. Bioanal. Chem..

[33] Morisset D., Štebih D., Milavec M., Gruden K., Žel J. (2013). Quantitative analysis of food and feed samples with droplet digital PCR. PLoS ONE.

[34] Jones G.M., Busby E., Garson J.A., Grant P.R., Nastouli E., Devonshire A.S., Whale A.S. (2016). Digital PCR dynamic range is approaching that of real-time quantitative PCR. Biomol. Detect. Quantif..

[35] Li J., Zhai S., Gao H., Xiao F., Li Y., Wu G., Wu Y. (2021). Development and assessment of a duplex droplet digital PCR method for quantification of GM rice Kemingdao. Anal. Bioanal. Chem..

[36] Zhu X., Liu P., Lu L., Zhong H., Xu M., Jia R., Su L., Cao L., Sun Y., Guo M. (2022). Development of a multiplex droplet digital PCR assay for detection of enterovirus, parechovirus, herpes simplex virus 1 and 2 simultaneously for diagnosis of viral CNS infections. Virol. J..

[37] He Z., Wang J., Fike B.J., Li X., Li C., Mendis B.L., Li P. (2021). A portable droplet generation system for ultra-wide dynamic range digital PCR based on a vibrating sharp-tip capillary. Biosens. Bioelectron..

[38] Shum E.Y., Lai J.H., Li S., Lee H.G., Soliman J., Raol V.K., Lee C.K., Fodor S.P.A., Fan H.C. (2022). Next-generation digital polymerase chain reaction: High-dynamic-range single-molecule DNA counting via ultrapartitioning. Anal. Chem..

[39] Fittipaldi M., Nocker A., Codony F. (2012). Progress in understanding preferential detection of live cells using viability dyes in combination with DNA amplification. J. Microbiol. Methods.

[40] Takahashi H., Gao Y., Miya S., Kuda T., Kimura B. (2017). discrimination of live and dead cells of *Escherichia coli* using propidium monoazide after sodium dodecyl sulfate treatment. Food Control.

[41] Papanicolas L.E., Wang Y., Choo J.M., Gordon D.L., Wesselingh S.L., Rogers G.B. (2019). optimisation of a propidium monoazide based method to determine the viability of microbes in faecal slurries for transplantation. J. Microbiol. Methods.

[42] Codony F., Dinh-Thanh M., Agustí G. (2020). key factors for removing bias in viability PCR-based methods: A review. Curr. Microbiol..

[43] Beer S., Norelli J. (1977). Fire blight epidemiology: Factors affecting release of *Erwinia amylovora* by cankers. Phytopathology.

[44] Biggs A.R. (1994). Characteristics of fire blight cankers following shoot inoculations of three apple cultivars. Acta Hortic..

[45] Kielak K., Sobiczewski P., Pulawska J. (2002). Overwintering of *Erwinia amylovora* in naturally and artificially infected appie shoots. Acta Hortic..

[46] Ordax M., Marco-Noales E., López M.M., Biosca E.G. (2006). Survival strategy of *Erwinia amylovora* against copper: Induction of the viable-but-nonculturable state. Appl. Environ. Microbiol..

[47] Ordax M., Biosca E.G., Wimalajeewa S.C., López M.M., Marco-Noales E. (2009). Survival of *Erwinia amylovora* in mature apple fruit calyces through the viable but nonculturable (VBNC) state. J. Appl. Microbiol..

[48] Biosca E.G., Santander R.D., Ordax M., Marco-Noales E., Águila B., Flores A., López M.M., Mendez-Vilas A. (2009). Survival of *Erwinia amylovora* in rain water at low temperatures. Current Research Topics in Applied Microbiology and Microbial Biotechnology.

[49] Santander R.D., Catalá-Senent J.F., Ordax M., Flores A., Marco-Noales E., Biosca E.G., Mendez-Vilas A. (2010). Evaluation of flow cytometry to assess *Erwinia amylovora viability* under different stress conditions. Microorganisms in Industry and Environment.

[50] Santander R.D., Català-Senent J.F., Marco-Noales E., Biosca E.G. (2012). in planta recovery of *Erwinia amylovora* viable but nonculturable cells. Trees.

[51] Santander R.D., Oliver J.D., Biosca E.G. (2014). cellular, physiological, and molecular adaptive responses of *Erwinia amylovora* to starvation. FEMS Microbiol. Ecol..

[52] Santander R.D., Biosca E.G. (2017). *Erwinia amylovora* psychrotrophic adaptations: Evidence of pathogenic potential and survival at temperate and low environmental temperatures. PeerJ.

[53] Choi H.J., Kim Y.J., Park D.H. (2022). extended longevity of *Erwinia amylovora* vectored by honeybees under in vitro conditions and its capacity for dissemination. Plant Pathol..

[54] Barcina I., Arana I. (2009). The viable but nonculturable phenotype: A crossroads in the life-cycle of non-differentiating bacteria?. Rev. Environ. Sci. Biotechnol..

[55] Oliver J.D. (2010). recent findings on the viable but nonculturable state in pathogenic bacteria. FEMS Microbiol. Rev..

[56] Pinto D., Santos M.A., Chambel L. (2015). thirty years of viable but nonculturable state research: Unsolved molecular mechanisms. Crit. Rev. Microbiol..

[57] Sobiczewski P., Pulawska J., Berczyński S., Konicka M. (1999). Fire blight detection and control in Poland. Acta Hortic..

[58] Sobiczewski P., Kielak K., Puławska J., Berczynski S. (2006). Winter survival of *Erwinia amylovora* in apple terminal shoot tissue. Phytopathol. Pol..

[59] Sobiczewski P., Iakimova E.T., Mikiciński A., Węgrzynowicz-Lesiak E., Dyki B. (2017). Necrotrophic behaviour of *Erwinia amylovora* in apple and tobacco leaf tissue. Plant Pathol..

[60] Ordax M., Piquer-Salcedo J.E., Santander R.D., Sabater-Muñoz B., Biosca E.G., López M.M., Marco-Noales E. (2015). Medfly *Ceratitis capitata* as potential vector for fire blight pathogen *Erwinia amylovora*: Survival and transmission. PLoS ONE.

[61] Grey B.E., Steck T.R. (2001). the viable but nonculturable state of *Ralstonia solanacearum* may be involved in long-term survival and plant infection. Appl. Environ. Microbiol..

[62] Mock N.M., Baker C.J., Aver’yanov A.A. (2015). induction of a viable but not culturable (VBNC) state in some *Pseudomonas syringae* pathovars upon exposure to oxidation of an apoplastic phenolic, acetosyringone. Physiol. Mol. Plant Pathol..

[63] Szatmári Á., Móricz Á.M., Schwarczinger I., Kolozsváriné Nagy J., Alberti Á., Pogány M., Bozsó Z. (2021). A pattern-triggered immunity-related phenolic, acetosyringone, boosts rapid inhibition of a diverse set of plant pathogenic bacteria. BMC Plant Biol..

[64] Venisse J.-S., Gullner G., Brisset M.-N. (2001). evidence for the involvement of an oxidative stress in the initiation of infection of pear by *Erwinia amylovora*. Plant Physiol..

[65] Venisse J.-S., Barny M.-A., Paulin J.-P., Brisset M.-N. (2003). Involvement of three pathogenicity factors of *Erwinia amylovora* in the oxidative stress associated with compatible interaction in pear. FEBS Lett..

[66] Santander R.D., Monte-Serrano M., Rodríguez-Herva J.J., López-Solanilla E., Rodríguez-Palenzuela P., Biosca E.G. (2014). exploring new roles for the *rpoS* gene in the survival and virulence of the fire blight pathogen *Erwinia amylovora*. FEMS Microbiol. Ecol..

[67] Santander R.D., Figàs-Segura À., Biosca E.G. (2018). *Erwinia amylovora* catalases KatA and KatG are virulence factors and delay the starvation-induced viable but non-culturable (VBNC) response. Mol. Plant Pathol..

[68] Morcia C., Ghizzoni R., Delogu C., Andreani L., Carnevali P., Terzi V. (2020). Digital PCR: What relevance to plant studies?. Biology.

